# Quality of Life and Unmet Need in People with Psychosis in the London Borough of Haringey, UK

**DOI:** 10.1100/2012/836067

**Published:** 2012-11-19

**Authors:** Maria Lambri, Apu Chakraborty, Gerard Leavey, Michael King

**Affiliations:** ^1^The Royal Society of Medicine, 1 Wimpole Street, London W1G 0AE, UK; ^2^Squamish Mental Health and Addictions, 38075 2nd Avenue, Squamish, BC, Canada V8B 0C2; ^3^Bamford Centre for Mental Health and Wellbeing, University of Ulster, Londonderry BT48 7JL, UK; ^4^University College London, Gower Street, London WC1E 6BT, UK

## Abstract

*Objectives*. Deinstitutionalization of long-term psychiatric patients produced various community-based residential care facilities. However, inner-city areas have many patients with severe mental illness (SMI) as well as deprivation, unemployment, and crime. This makes meeting their community needs complex. We undertook a needs assessment of service provision and consonance between service users' evaluation of need and by care workers. *Design*. Cross-sectional study with random sample of SMI service users in four housing settings: rehabilitation units; high-supported; medium-supported; low-supported housing. *Setting*. London Borough of Haringey. *Outcome Measures*. 110 SMI service users and 110 keyworkers were interviewed, using Camberwell Assessment of Need; SF-36; Lancashire Quality-of-Life profile; demographic and clinical information. *Results*. People in “low-support” and “high-support” housing had similar symptom scores, though low support had significantly lower quality of life. Quality of life was positively predicted by self-reported mental-health score and negatively predicted by unmet-need score in whole sample and in medium-support residents. Residents' and care-workers' assessments of need differed considerably. *Conclusions*. Although patients' housing needs were broadly met, those in low-supported housing fared least well. Attendance to self-reported mental health and unmet social needs to quality of life underpins planning of residential services for those with SMI. Social and personal needs of people in supported housing may be underestimated and overlooked; service providers need to prioritise these if concept of “recovery” is to advance.

## 1. Introduction

### 1.1. Background

An important theme to emerge over the last two decades since the move away from institutional care and towards community mental health care services has been the recognition of the importance of a needs-led approach towards care provision for the individual with severe mental illness (SMI). Housing and socioeconomic care needs form the central thrust to this approach, acting as stabilising forces to establish a daily routine and address life issues. However, due to low income, stigma, difficulties in daily functioning inherent to SMI, and fluctuations in symptoms, people with SMI find it difficult to compete for better-quality housing and often live in substandard accommodation that is physically inadequate, crowded, noisy, and located in noisy neighbourhoods [1].

This suggests a fundamental mismatch in meeting the needs of those with SMI. A prospective needs study across six European countries of those with schizophrenia found that one in four patients had needs that were not adequately met by the mental health service in their region [2]. It also found a systematic relationship between the availability of community-based mental health care and the need status of its cohort: the fewer outpatient and rehabilitation services available, the more unmet needs there were. 

### 1.2. Housing and Well-Being

Planned housing support is central to a mental health promotion strategy, helping to reduce the incidence and prevalence of mental illness and unnecessarily long stays in hospital settings; it also makes good fiscal sense. The shift away from a response to homelessness that focuses on providing emergency services to one that emphasizes prevention can, if implemented effectively, save money, according to a Toronto-based report [3]. Prevention means stopping people from becoming homeless in the first place. An example of this is improving discharge planning and transitional housing (and supports) for people leaving hospital. By providing supports to someone who would otherwise become homeless the life-time savings to the system are considerable.

The provision of affordable, decent-quality, adequately supportive accommodation is a major factor in helping people recover from mental illness and decreasing the risk of depression, hospitalisation, suicide, family break-up [4, 5]. However, a recent systematic review of studies examining the effect of housing need on health, quality of life, and healthcare use for those with SMI reported that there is a dearth of evidence of housing solutions for those with SMI in precarious or unsupported housing [6].

The deinstitutionalization of long-term psychiatric patients has led to the creation of a wide variety of community-based residential care facilities. In designing such residences, “a balance must be sought between providing structure and protection on the one hand and fulfilling the aims of normalization and community integration on the other” [7]. This tension in mental health care has been highlighted and underpinned by a growing interest in recovery models, empowerment, and social inclusion [8, 9].

### 1.3. Supported Housing

Supported housing may be provided by private and voluntary sectors, the statutory sector, housing associations and charitable organisations. Briefly, models of supported accommodation include communal group homes and hostels with onsite support workers; therapeutic communities; independent living supported housing schemes for people with mental health problems through self-contained accommodation located in one building or site, with onsite support workers during office hours; independent tenancies in general needs housing with outreach workers or floating support visits regularly. 

Evidence suggests high levels of satisfaction with supported housing amongst patients and relatives compared to hospital environments [10], an improvement in social functioning, a dramatic reduction in hospital admissions, higher levels of social networks, and a reduced level of the negative symptoms of schizophrenia. There is also a decrease in the rates of subsequent homelessness, other psychiatric symptoms [11], and overall cost [12]. This, however, may be at the risk of increasing dependence on professionals and prolonging exclusion from the community [13]. 

Some patients, moreover, have concerns regarding the stigma and restrictiveness of such high levels of supported accommodation, including boredom and having poor access to leisure and recreational facilities. Similarly, community living does not always equate with increased patients' social networks. There have also been reports in bias in the selection of patients for placements leaving the most disturbed individuals in hospital environments [4].

### 1.4. Need in Haringey

According to the 2001 census Haringey has a population of over 216,000, ranking it the 50th most dense district in the United Kingdom. However, it is considered that the census may have underestimated the population of the borough. There is a clear divide between the affluent west of the borough, which include the wards of Highgate, Muswell Hill, Crouch End, and Alexandra, and the east of the borough which has considerable levels of deprivation: 40% of residents live in wards that are amongst the 10% most deprived in the UK. These include White Hart Lane, Northumberland Park, Noel Park, Bruce Grove, Tottenham Hale, Tottenham Green, Haringey, and Hornsey (index of deprivation score is 50.3–66.4 and depicts most deprived quintile). With the strengths of its multicultural environment, the borough representing over 50% of its population from ethnic minorities, there are also challenges that the community face. Associated with the levels of deprivation are high rates of long-term unemployment, mental and physical ill-health, substance misuse, crime, asylum seekers, and large numbers of homeless households. Many of these households live in insecure temporary housing thus making efforts from the local authority and allied service agencies to promote social inclusion and cohesion more complex. 

Haringey exhibits a high level of SMI in its population. An inpatient census was carried out by BEH MHT in August 2004 and identified that the most frequent diagnosis of those admitted was schizophrenia (35%). When all the psychotic disorders were grouped (schizoaffective disorder, psychosis, bipolar disorder, and schizophrenia), this accounted for 55% of those admitted.

The mental illness needs index [14] records need for specialist mental health services for SMI. It incorporates population characteristics, which contribute to variation in hospital admissions, indicators of deprivation, long-term illness, and disability, and the numbers of people living in a hostel/lodging house. A score of more than 1.0 represents a greater need for mental health services. The model suggests that the need for mental health services in Haringey is 1.16, similar to the London average of 1.15. Some wards in Haringey, namely, Seven Sisters, Noel Park, Bruce Grove, and Northumberland Park, however, have a greater need for mental health services with scores ranging from 2.01–2.33, which is twice the national average.

### 1.5. Need, Functioning, and Quality of Life

When evaluating needs and the policy of deinstitutionalization by comparing hospitalized and community residents, extensive research has shown the importance of a combined evaluation of functioning, clinical status, individual needs assessments, and quality of life to inform service provision. 

A broad definition of quality of life is “adequate resources, fulfilment of social roles in multiple life domains, satisfaction with life in various domains, and general life satisfaction [15].” People with SMI report key problem areas that affected their quality of life were: a lack of personal achievement, lack of job, difficulty in forming and maintaining relationships, loneliness, health problems (both mental and physical), lack of leisure activities, personal safety, and looking after themselves. More often than not, individuals with SMI generally display lower levels of educational, financial, and vocational achievement than the general population. 

Identifying unmet needs can provide information for gaps in services and implications for improvement. Evidence from Slade and colleagues [16] and the UK700 Group [17] showed that meeting unmet need was important because the number of unmet needs was related to reduced health and ongoing health-related expenses. Also, Slade and colleagues [18] observed that as needs increase, quality of life decreases and that unmet needs have more influence on quality of life than met needs. 

Quantifying functioning and identifying need at a local level for people with SMI will enable providers and patients to access a range of different forms of supported accommodation, through which patients may move according to needs as well as by choice, at different times in their lives or stage of their illness.

### 1.6. Haringey SP Programme

The Haringey “Supporting People” (SP) programme provides 377 units of housing-related support (HRS) for people with mental health problems in the borough. The level of HRS offered varies from very low levels of provision to very intensive services. Although the Local Authority administers the programme, decisions on commissioning and strategy are made by a partnership including the Local Authority, the Primary Care Trust and Probation. 

This study was exploratory in nature and sought to collect information from a random sample of those with SMI residing in different housing types in Haringey. Demographic and clinical data was collected in order to form a profile of residents in different housing, as well as to measure their degree of met and unmet need. We aimed to examine differences between the self-assessed needs of residents and those determined by their caseworkers. Additionally, we sought to determine which variables (if any) predicted quality of life, irrespective of housing type.

## 2. Methods

### 2.1. Participants

110 participants with severe mental illness (SMI), and 110 keyworkers were interviewed. Inclusion criteria were (1) a lower age limit of 16 years, with no upper age limit; (2) a primary diagnosis of SMI; defined as a clinical diagnosis of schizophrenia, bipolar disorder, or other psychosis made by the psychiatrist in-charge; (3) to be resident in the Rehabilitation Units at St Ann's Hospital, high-support-accommodation (24 hr staffing including waking night staff), medium-support accommodation (staff available during the whole day or visiting regularly), or low support accommodation (peripatetic staff and/or an alarm or on-call system) within the London Borough of Haringey. Accommodation grading was based on the information provided by the local authority (LA) on the housing providers' service provision. This was subsequently grouped according to the GLA reports criteria [19]. Participants with a primary diagnosis of substance misuse or an organic condition were excluded.

### 2.2. Procedure

Housing providers in Haringey were identified through the LA database. The aim was to include a representative sample covering residential care to low level supported housing, with a minimum of a third from each of the residences/providers. A sample of participants was identified through the LA database and local patient register for potential inclusion. The approach was fully compliant with the Data Protection Act. A random sample of those who were interested was selected from the database for the purposes of the needs-assessment interview. Of those individuals who refused or were ineligible, the next person on the list was chosen. 

The interview process involved validated survey methods using face-to-face interviews at the individual's own home unless they requested otherwise, at a time convenient to the participants and following the safety guidelines for researchers. Participants received *£*10 expenses for their involvement. Individual keyworkers were interviewed separately to complete the test battery. Data was triangulated by gathering additional information from the participants' keyworker and medical and other case notes. All participants completed a written informed consent form. Verbal consent was obtained from keyworkers. 

### 2.3. Measures

All participants were evaluated using the following validated instruments.

#### 2.3.1. The 36-Item Short Form (SF-36) [20]

It was constructed to survey health status in the Medical Outcomes Study. It is a generic measure, which can be interviewer or self-administered, and assesses eight health concepts (physical functioning; physical role; bodily pain; general health; vitality; social functioning; emotional role; mental health). The scores range from 0–100, with a higher score indicating a greater level of functioning. 

#### 2.3.2. Camberwell Assessment of Need Research (CAN-R) [21]

The instrument assesses needs for care and help over the last month in 22 health and social domains. Separate assessments can be recorded from the perspectives of the service user and staff. CAN-R has four sections for each of the 22 domains assessed: existence of a need; informal help; help from formal services; user satisfaction with help. Based on responses, a “need rating” is made for the last month within each domain by means of a three-point scale. The number of met and unmet needs may be scored per domain. An overall total need score can also be summed. 

#### 2.3.3. Lancashire Quality of Life Profile [22]

This is an interviewer-administered questionnaire based on Lehman's work and retains eight of Lehman's domains (health and self-concept, social relations; law/safety; living situation; leisure/participation; family; work; and finances; religion). Objective ratings are made from direct questioning about participant's lives, while subjective well-being is measured by asking participants to rate their satisfaction with each separate life domain on a seven-point Likert scale.

#### 2.3.4. Brief Psychiatric Rating Scale: Expanded Version 4.0 [23]

This is an interviewer-based instrument and rates the severity of psychiatric symptoms on a scale of one to seven (not present to extremely severe) in 24 domains. Information is also gathered via behavioural observation, medical case notes, and from keyworker. The BPRS contains four symptom clusters that tend to cooccur. 

#### 2.3.5. WHO Life Chart

This consisted of health-related information gathered from medical notes charting the past two years [24].

### 2.4. Statistical Analysis

Basic descriptive statistics of the sociodemographic and clinical were calculated for the whole sample and for all four groups of supported accommodation residents. These data were also examined in relation to assessment of need as determined by participant's scores on the SF-36, the CAN-R, and Lancashire Quality of Life Profile domain scores and overall scores. 

Next, regression analysis was used to model the effect of need upon quality of life, therefore, the primary exposure was “need”, and the primary outcome was “quality of life”. The following list of *a priori* variables was compiled by the Research Group (with expertise in the field), and considered for confounding: age, gender, ethnicity, BPRS score, SF-subscore, medication, suicidality, and hospital admissions. Prior to analysis, variables were checked for normality of distribution. If they were not normally distributed, they were collapsed into categories to make maximal use of the information. Those confounders found to be significantly correlated with either the primary exposure or primary outcome, were then entered into each regression analysis. Analysis was performed for the whole sample and by housing group, separately.

## 3. Results

The demographic and clinical data of the participant sample is shown in Table 1. The sample's mean age was 41.6 years and the majority were male (77.3%), single (79.1%), of African-Caribbean ethnicity, and diagnosed with schizophrenia (81.8%). The greatest proportion interviewed resided in “medium-supported” housing (staff was available and visited but not permanently on site). Total psychiatric symptom score measured on the Brief Psychiatric Rating Scale (BPRS) was ten points higher in rehabilitation than other housing types, suggesting greater symptomatology in this group. 

Table 2 summarises self-reported health scores for the sample measured by the SF-36, with a higher score representing greater functioning and ability. There were subtle differences between housing types, but one trend is evident: the highest scores were seen in those in high-supported accommodation, with the lowest scores mostly seen in low-supported residents. Physical functioning and role limitation were reported as poorest in rehabilitation residents. Interestingly, those in rehabilitation reported the greatest mental health scores, despite having the highest BPRS totals (see Table 1).

Need scores perceived by participants and their keyworkers measured using the CAN are shown in Table 3, above. There was significant dissonance in all-need scores between participant and keyworker in all housing settings apart from in rehabilitation units. More met needs were reported by keyworkers than by participants in all settings, except in low-supported housing. The met-need score tended to increase from low-supported accommodation through rehabilitation settings; however, the unmet-need score varied far less across housing types.

Quality of life (QoL) scores are reported in Table 4. Overall, differences were minimal. Participants in high-supported accommodation had the greatest total QoL score as well as the greatest number of highest scores, whereas the total QoL score was lowest in low-supported residents.

Table 5 shows the results of multiple regression with quality of life as the outcome; only significant exposure variable was included in the final models. Model one was performed for the entire sample. It showed that a greater Lancashire quality of life score was predicted by lower user-determined unmet needs (*b* = −2.20) and greater SF-36 mental scores (*b* = 0.029). Model two was performed for the largest subsample of participants: those in medium-supported housing. Model one's findings were replicated: quality of life was predicted by lower user-determined unmet needs (*b* = −0.27) and greater SF-36 mental score (*b* = 0.016).

## 4. Discussion

### 4.1. Summary of Findings

This was a cross-sectional study of a random sample of people with psychosis residing in various types of housing within the London Borough of Haringey. The main findings were as follows: the largest ethnic group was of African-Caribbean origin and the largest housing group resided in medium-supported accommodation (where trained staff were available during the day or visited regularly). Those interviewed in the rehabilitation units, who received the greatest level of trained support, also exhibited the greatest observer-rated psychiatric symptom scores but scored themselves least for symptoms. 

In community residences, those in low-support accommodation provided lower scores than those in high-support housing for the various domains of social functioning measured by the SF-36, despite similar levels of psychopathology in both environments. This was also reflected in quality of life scores, which were commensurate with level of support provided.

Within the needs assessment, the magnitude of unmet need was less than that for met need, and this did not vary across housing type. Finally, self-reported mental health and unmet need were the only significant variables to predict quality of life score in regression analyses of the sample as a whole and those in medium-support housing alone. 

### 4.2. Limitations of the Study

This was a cross-sectional study, which makes it difficult to infer causality between variables that are associated with each other; a prospective study would make aetiological inferences easier. Although the study sample was selected in a random manner, there may have been a systematic bias in those that agreed to be interviewed. However, the number that refused to participate was minimal which limits this possibility. Finally, the smaller subsamples in rehabilitation, high- and low-support housing may have limited precision and masked any significant effects in the regression analyses. However, the overall housing profile of those interviewed is likely to reflect the real-life population where most people on the Haringey register with SMI are in medium-support housing.

### 4.3. Implications

Need scores tended to be greater when estimated by keyworkers rather than the participants themselves. This is reflected in other research findings [25] and may represent a degree of concern by service users not to overstate their perceived needs to figures in authority in case it led to overly restrictive accommodation interventions. This larger estimation of need by keyworkers may also represent a deliberate way of ensuring that the users' needs are adequately met, thereby reflecting a general concern that resources available to clients are limited and in demand.

We found that the magnitude of unmet need was less than for met need, irrespective of housing type, implying the relatively successful and important function of residential facilities for those with SMI. This may be a global and time-independent finding as it has been replicated in a recent Indian study using the CAN of patients with SMI living in a half way home in Bangalore [26]. 

Those in high-support accommodation tended to show higher social function scores and self-perceived quality of life scores than those in low-support housing. Another UK study found that patients who moved from more independent living to a group home showed both a reduction in psychiatric symptom scores and concomitant improvement in their quality of life scores [27]. Baker and Douglas in the USA noted that a move from housing that was appropriate to need to more inappropriate housing led to deterioration in quality of life of those with SMI [28]. It is important to note that only trends in scores were found rather than significant differences: a Canadian study revealed no differences in mean quality of life scores between those with SMI in supportive housing and those in basic “board-and-care” homes [29]. Nevertheless, these trends may have become more significant with a larger sample size.

Our regression analyses demonstrated that self-reported mental health score was positively predicted, and unmet-need score was negatively predicted quality of life. Lasalvia and colleagues also found that self-reported psychological distress rather than BPRS score predicted subjective quality of life [30]. In terms of need, the same-led research group reported upon a four-year cohort study which found that an improvement in clinical conditions, and a reduction in unmet-need predicted a lower follow-up quality of life, suggesting self-perceived social needs rather than reduction in psychopathology-improved quality of life in those with SMI [27]. Additionally, a cross-sectional multicentred Nordic study of 418 schizophrenics found that more unmet needs were associated with a poorer quality of life, accounting for 6% out of 41% of the explained variance in quality of life at regression [31]. This highlights the importance to attend to (and hopefully improve) the subjective experience of illness and distress as well as addressing the social needs of those with SMI.

### 4.4. Conclusions and Future Research

The needs assessment of this sample found the mean unmet-need scores to be lower than met needs suggesting that needs are being broadly met for those with SMI in Haringey. However, those in low-support housing seemed impaired and distressed by the relative lack of input they received, reflected in their lower social-functioning and quality of life scores. 

The lack of variation in need scores across housing types may suggest that the apportioning of housing may be more arbitrary than previously believed. An interesting study would be to follow up those with SMI in low-support housing and see if their perceived disadvantage is reflected in increased hospital admission. 

Another important point was that observer-rated psychiatric symptom score did not determine quality of life, although self-reported psychopathology and social need did. The aetiological significance could be underscored by a prospective study in Haringey, as performed by Lasalvia's group in Italy [32]. If this were the case, it could steer the emphasis of community services for those with SMI more toward relieving the individual's subjective experience of distress.

Finally, that needs are often not being met should not be lost in this study's findings. It may reflect a global problem of inadequately meeting the needs of those with mental illness in general, as has been seen at the population level in Europe [33]. It may also indicate that needs are interdependent, with the failure to meet a need in one domain, having a detrimental effect on need in other areas. A recent study by colleagues in Buffalo, New York highlighted the problem of the unmet need of social connectedness of patients with SMI [34]. This acted as an obstacle to the global goal of recovery—patients returning to or achieving meaningful social roles, relationships, and membership in their communities; it also indicated that needs may be “cantilevered”, rather than hierarchical, with each essential to the other and to overall functioning and well-being. Finally, another population survey of Swedish mental health burden found that those most likely to have an unmet need were males, socially isolated, and had educationally underachieved [35]—descriptive factors that often describe those suffering with severe and enduring mental illness, irrespective of geographical origin. 

If we are to enable those with SMI in their process of recovery and reintegration, helping in the fulfilment of need should be paramount.

## Figures and Tables

**Table 1:** Demographic and clinical data, by housing type (*n* = 110).

Participant characteristic	Rehab (inpatient)		High support (including waking night staff)		Medium support (staff available during whole day/visit regularly)		Low support (peripatetic staff and/or alarm/on-call)	
	*n *	(%)	*n *	(%)	*n *	(%)	*n *	(%)
Age group								
16–25	—		2	(6.7)	4	(7.7)	3	(18.8)
26–35	2	(16.7)	3	(10)	15	(28.8)	3	(18.8)
36–45	3	(25)	10	(33.3)	20	(38.5)	6	(37.5)
46–55	4	(33.3)	10	(33.3)	9	(17.3)	3	(18.8)
56–65	—		5	(16.7)	4	(7.7)	1	(6.3)
66+	3	(25)	—		—		—	
Gender								
Male	10	(83.3)	24	(80)	41	(78.8)	10	(62.5)
Female	2	(16.7)	6	(20)	11	(21.2)	6	(37.5)
Ethnicity grouped								
African Caribbean	7	(58.3)	17	(56.7)	29	(55.7)	10	(26.6)
White British	3	(25)	6	(20)	11	(21)	5	(31.3)
European	1	(8.3)	4	(13.3)	10	(19.2)	1	(6.3)
South Asian	1	(8.3)	3	(10)	2	(3.8)		—
Time in residence, grouped								
0–12 months	2	(16.7)	8	(26.7)	14	(26.9)	6	(37.5)
13–28.5 months	3	(25.0)	4	(13.3)	13	(25.0)	5	(31.3)
28.6–48 months			9	(30.0)	18	(34.6)	4	(25.0)
>49 months	7	(58.3)	9	(30.0)	7	(13.5)	1	(6.3)

BPRS scores	Mean	S.D.	Mean	S.D.	Mean	S.D.	Mean	S.D.

Total	64.83	7.41	54.03	14.29	52.29	12.54	52.0	11.46
Psychotic index	11.08	2.39	7.70	3.78	6.48	2.66	7.44	3.29
Prodromal score	12.08	3.09	9.70	3.46	11.08	3.02	11.13	2.83
Anxious depression	6.50	2.78	5.37	2.48	6.54	2.77	7.13	2.99
Withdrawal retardation	8.33	2.27	7.67	2.95	7.15	2.63	6.94	2.82
Hostile suspiciousness	8.92	3.20	6.03	2.87	6.42	2.52	7.50	2.25

**Table 2:** Comparison of mean self-reported health scores (SF-36), across housing type.

SF-36 summary scores	Rehab settings	High-support	Medium-support	Low-support
		accommodation	accommodation	accommodation
Physical functioning	66.3	82	81.5	76.6
Role limitations due to physical problems	59.1	75	76.9	68.8
Role limitations due to emotional problems	61.1	73.3	59.6	56.3
Bodily pain	74	75.1	68.3	67.2
General health	64.7	65.5	59.3	56.3
Vitality	55.5	59.6	51.5	47.2
Social Functioning	82.5	97.4	89	79.3
Mental health	74.8	71.4	63.3	60.5

**Table 3:** Comparison of the mean total needs, met and unmet needs in all housing types (Wilcoxon-matched pairs signed-rank test).

CAN Score	Keyworker	Participant	Difference	*P* value
	Mean (S.D.)	Mean (S.D.)		
Rehab				
All needs	10.6 (2.0)	9.7 (2.0)	0.9	0.44
Met needs	8.8 (1.5)	7.5 (1.4)	1.3	0.05
Unmet needs	1.8 (1.1)	2.2 (1.9)	0.5	0.60
High support				
All needs	9.1 (2.5)	7.8 (2.2)	1.3	0.006
Met needs	7.3 (2.1)	6.7 (2.0)	0.6	0.07
Unmet needs	1.8 (1.6)	1.1 (1.2)	0.7	0.70
Medium support				
All needs	8.2 (3.2)	7.3 (2.7)	0.9	0.07
Met needs	6.8 (2.7)	5.8 (2.3)	1.0	0.02
Unmet needs	1.6 (1.6)	1.5 (1.5)	0.1	0.80
Low support				
All needs	5.9 (3.1)	6.5 (2.9)	0.6	0.006
Met needs	4.4 (1.6)	4.8 (2.4)	0.4	0.2
Unmet needs	1.6 (1.9)	1.7 (1.1)	0.1	0.7

**Table 4:** Lancashire quality of life scores across housing type.

	Housing type							
	Rehab (inpatient)		High-support 24 hr staffing inc. waking night staff. res.		Med-support staff available during whole day/visit reg. (SP)		Low-support peripatetic staff and/or alarm/on-call (SP)	
	Mean	Std deviation	Mean	Std deviation	Mean	Std deviation	Mean	Std Deviation
LQoL general well-being score	3.91	1.83	4.86	1.30	4.70	1.22	4.72	1.90
LQoL mean education score	4.40	1.26	4.24	1.64	3.88	1.38	4.03	1.34
LQoL leisure score	4.64	1.65	5.21	1.06	5.07	.88	4.60	1.09
LQoL religion score	4.90	.74	5.15	.69	4.87	1.05	4.17	1.11
LQoL finances score	3.96	1.47	4.70	1.06	4.44	1.16	4.16	1.50
LQoL living situation	3.94	1.25	4.87	1.06	4.79	.91	4.74	1.19
LQoL legal and safety score	5.55	2.24	5.22	1.09	4.82	1.01	4.88	1.36
LQoL family relations score	4.88	1.52	4.97	1.36	4.84	1.49	4.59	1.67
LQoL social relation score	4.50	1.35	4.92	1.03	4.81	.85	4.91	.97
LQoL health score	4.75	1.23	4.94	.99	4.74	.91	4.75	1.07
LQoL total score	4.59	.65	4.90	.56	4.68	.58	4.57	.87

**Table 5:** Significant models following multiple regression analysis with general quality of life as the primary outcome.

Primary outcome	Coefficient	S.E.	*t*	*P* > |*t*|	[95% CI]
General quality of life					
Exposure variables:					
Model one (all housing)					
User's unmet needs	−2.20	−0.089	−2.29	0.025	−0.38, −0.03
SF-36 mental score	0.027	0.005	5.29	0.000	0.017, 0.038
Constant	3.29	0.40	8.13	0.000	2.49, 4.09

Model two (med support)					
User's unmet needs	−0.27	0.10	−2.61	0.012	−0.47, −0.06
SF-36 mental score	0.016	0.0065	2.51	0.016	0.0032, 0.030
Constant	4.11	0.50	8.30	0.000	3.11, 5.11
